# Supplementary material for: Are carbon emissions trading and green financial instruments synergistic? -Comprehensive quantitative research based on content analysis
Source: PLoS One. 2024 Mar 7;19(3):e0298601. doi: 10.1371/journal.pone.0298601 (PMC10919601; doi:10.1371/journal.pone.0298601)
Supplement: S1 Data — (ZIP) [file pone.0298601.s001.zip › Data/地级市Z_F工作报告.zip!/┴╔─■/│»╤⌠╩╨/└┤╫╘╣1⁄2╓┌║┼í╛┬φ┐╦╩2╛▌═°í┐_2017.docx]

各位代表：

　　现在，我代表朝阳市人民政府向大会报告工作，请予审议，并请各位政协委员和其他列席人员提出意见。

2016年工作回顾

　　刚刚过去的一年，面对经济下行和修复政治生态的双重压力，我们在省委、省政府和中共朝阳市委的坚强领导下，在市人大及其常委会和市政协的监督支持下，认真贯彻落实五大发展理念和“四个着力”要求，以推进供给侧结构性改革为主线，迎难而上，奋力拼搏，取得了来之不易的成绩。

　　去年下半年起，我们统一认识，定下决心，做实经济数据。预计全年完成地区生产总值780亿元，下降9%；规模以上工业增加值下降18%；公共财政预算收入51亿元，下降6.8%；固定资产投资243亿元，下降51.4%；社会消费品零售总额447亿元，增长4.5%；城镇和农村常住居民人均可支配收入分别为22500元和11260元，增长6.1%和7.1%。

　　过去一年，经过不懈努力，一批对朝阳发展具有深远影响的重大政策、重大项目和重点工作取得了新突破。一是五个县级工业园区获省政府一次性批复，全部晋升为省级经济开发区，总量仅次于沈阳市，与大连市并列第二位。二是我市被纳入国家行政审批制度改革试点，并在全省率先组建了行政审批局和市场监管局。三是强力开展矿山矿业整治，对隐患多、污染重以及违规开采的小矿山进行依法治理，铁选企业、采石场分别压缩到47家和37家。四是重拳治理燃煤小锅炉、淘汰黄标车和老旧汽车，实施秸秆禁烧，清理整顿环保违规项目，全市空气质量达标天数比例达89%，全省排名第一。五是项目年取得丰硕成果，实施亿元以上建设项目167个，其中新开工94个，数量质量在辽西北地区乃至全省处于领先。六是环城生态绿廊、北部城区集中供热、辽西北供水配套设施启动建设，将极大提升城市品质和民生福祉，牵动未来发展。七是京沈高铁铺轨施工，沈朝段今年年底试运行，朝阳即将迎来“高铁时代”。

　　一年来，我们坚持一张蓝图绘到底，保持工作连续性稳定性，主要做了以下几个方面的工作：

　　一、全力以赴稳增长

　　“五个一工程”体制机制创新、融资平台搭建、基础设施建设、项目引进落地齐头并进，一批特色鲜明的项目集聚平台和增长点加快形成，全市近80%的新上项目和75%的固定资产投资来自各园景区。朝阳县中庆集团工业地产项目已建成标准厂房23万平方米，喀左浴龙谷温泉度假村和“产业大道”“旅游大道”等项目加快推进，北票台州汽摩配产业园启动建设，龙城老企业对外合资合作迈出新步伐，凌源航天凌河整车项目通过国家审批即将开工建设，建平、双塔园景区规划和建设也都有了新的进展。“五个一工程”的实施，为朝阳突破困境、实现振兴发展带来了转机、信心和希望！

　　全面贯彻落实中央7号文件，明确振兴发展十大重点任务，全年共争取中央预算内投资8亿元、国家专项建设基金36.8亿元、省产业（创业）投资引导基金直投项目1.4亿元。

　　支持凌钢、鞍钢朝阳钢铁公司、浪马等重点企业加快技改升级，加大停产半停产企业帮扶力度，积极盘活闲置土地和厂房，工业经济企稳趋势开始显现。强力开展项目要素保障百日集中行动，逐一梳理问题，推行实名制建档，为项目如期开复工做好准备。

　　主动对接京津冀协同发展战略，承接京津冀和东南沿海地区产业转移，组织开展7次大型招商活动，洽谈推进110多个重点项目，一批大企业大集团进驻朝阳。抢抓“一带一路”战略机遇，企业“走出去”和国际产能合作取得新进展，实现出口6.3亿美元。

　　二、坚定不移调结构

　　狠抓“三去一降一补”任务落实。50万吨钢铁、8万吨煤炭过剩产能化解和30万亩玉米调减任务全面完成。完成商品房销售105万平方米，通过棚改消化存量住房44.3万平方米。帮助企业拓宽融资渠道，新增融资135亿元，引进金融机构4家，新增各类股权交易市场挂牌企业6户。通过各种措施，减免企业税负3800万元，减轻企业负担4000万元，降低企业用电成本3720万元。

　　粮食生产喜获丰收，产量接近25亿公斤。实施各类节水灌溉工程24.9万亩，新增设施农业面积1.5万亩。启动农产品区域公用品牌创建工作，“朝阳蔬菜”“朝阳小米”品牌发展规划编制完成。农产品加工园区建设扎实推进，龙头企业达360家，实现销售收入210亿元。畜牧兽医工作获国家农业部表彰。

　　全面落实中国制造2025朝阳行动纲要。引进战略合资合作伙伴，促进现有企业转型升级，佛瑞达与北京骏豪新型包装产业园、辽宁新大新能源与西安隆基高效单晶硅、朝工与北京上唐新能源汽车等一批重点项目启动实施。加快新兴产业培育，天翼国基、立塬新能源、天佑紫砂、坤鹏矿联网等一批创新型企业快速成长，新增高新技术企业10户，创历年新高。

　　服务业增加值比重达到46%，提高4个百分点。万达广场、凤凰商城投入运营，中信化石产业园、天马时代广场进展顺利。农产品现代流通体系建设和肉菜流通追溯体系试点扎实推进。新兴业态蓬勃发展，阿里巴巴、京东等知名电商落户我市，电商企业突破100家，交易额突破40亿元，覆盖县乡村三级的农村电子商务体系初步成型。

　　大黑山与江西三清山日上、清风岭与吉林昊融、凤凰山与中合置业合资合作全面推进。鸟化石公园入选世界地质公园候选名单，喀左、凌源入选国家全域旅游示范区创建单位。

　　三、突出重点促改革

　　坚持市县同步，全域推进国家行政审批制度改革试点。按照大部制方向推进政府机构改革，市行政审批局、市场监管局、住建委、国资委、商务局、经合局等完成组建调整。机关公务用车制度改革顺利完成。

　　实施市直党政机关所属企业和经营性事业单位改革，市属40余户企业完成脱钩。组建成立国恒国有资本运营公司和水务、城发、文投3家企业集团，实现管资产向管资本转变。

　　农业共营制改革试点全面展开，推进试点合作社41家。农村土地确权颁证范围进一步扩大。供销社综合改革、国有林场和集体林产权制度改革扎实推进。

　　出台促进大众创业、万众创新政策，全面实施“五证合一、一照一码”登记制度改革，新增各类市场主体3.05万户、从业人员6万人，发放创业担保贷款5.22亿元。双创孵化基地面积已达7.8万平方米，龙城、喀左孵化基地分别被评为国家级和省级创业创新示范基地。10户企业在全省双创大赛上荣获奖项，健德“城市蜗牛”等4户企业代表辽宁参加全国大赛并取得佳绩。

　　四、积极稳妥防风险

　　规范政府债务管理，严控债务规模，当年到期和逾期政府债务全部置换，并提前置换了部分未到期债务。

　　加强养老金管理，通过强化基金征缴、多渠道筹集资金、调整支出结构等措施，保证了养老金按时足额发放。

　　加强财政预算执行和管理，优化支出结构，压缩一般性支出，保工资保运转保民生，严防财政支付风险。

　　强化社会稳定风险防控。持续开展隐患排查和整改，安全生产形势总体平稳。深入推进诉访分离、信访听证、网上信访，有效化解一批突出信访问题和历史积案。人民调解、社区矫正、法律援助取得新进展，综治维稳、严打整治、道路交通管理形成常态化，火灾防控能力不断提高。妥善应对两次集中强降雨，做到大雨大汛无大灾。全市村民委员会换届工作圆满完成。

　　五、竭尽所能惠民生

　　把脱贫攻坚作为最大最根本的民生工程来抓，逐级签订责任状，整合放大各种扶贫资金，为贫困户量身定做了产业扶贫项目，5.25万贫困人口实现脱贫，97个省级贫困村、25个市级贫困村摘帽销号。

　　实现城镇新就业3万人，城镇登记失业率3.8%。启动“五险合一”征缴模式，提高社保待遇标准，企业退休人员养老金月人均提高到2010元，城乡低保分别提高6%和14%，城镇居民医疗保险和新农合补助提高到420元。

　　持续推进青山、碧水、蓝天、沃土工程，完成荒山造林73.3万亩，水土保持综合治理110万亩；凌河流域水质稳定达标，沿河城乡防洪及生态景观建设取得新进展；积极推广农机深松整地和保护性耕作，深入开展农药执法检查，从源头减少面源污染。

　　完成棚改任务7278户，货币化安置率达83.1%。实施老旧住宅小区改造60万平方米、农村饮水安全巩固提升工程32处。市容环境集中整治深入开展，城市管理水平不断提升。宜居乡村建设、农村危房改造扎实推进，农村面貌进一步改观。

　　京沈高铁喀赤线开工建设，朝盘线获得批复，锦承线和叶赤线朝阳段扩能改造启动实施。朝巴线和文明路公铁立交实现通车。

　　新建改扩建标准化幼儿园13所，实施“全面改薄”项目学校116所。北票、凌源通过义务教育发展基本均衡县国家认定。高中阶段教育基本普及，现代职业教育体系加快形成。县级公立医院改革实现全覆盖，启动省内异地就医即时结算工作。市妇女儿童医院病房楼、公共卫生服务综合楼等项目顺利实施。乡镇综合文化站实现全覆盖，成功举办群众文化节、书香朝阳全民阅读活动。完成100处农民健身工程，朝阳籍运动员在里约获奥运会金牌1枚、残奥会金牌2枚。民族地区经济和各项事业又有新发展，宗教工作按新形势新要求得到改进和加强。国防动员、人防和民兵预备役建设工作扎实推进，我市再度荣获全国“双拥模范城”称号。气象、住房公积金、邮政、档案、应急管理、国家安全、援疆援青、外事侨务以及老年人、妇女儿童、残疾人权益保障等工作也取得了新成绩。

　　过去一年，我们自觉接受市人大及其常委会的法律监督和工作监督，办理代表议案2件、建议67件，问题解决率达70%，满意率100%；主动接受市政协民主监督，办理政协提案228件，采纳率提高4.5个百分点，满意率达99.6%。坚持全面从严治党，深入开展“两学一做”学习教育，认真落实巡视反馈意见整改，以辽宁拉票贿选案为镜鉴，净化政治生态，优化发展环境，加大行政监察、审计监督和督查督办力度，政府工作作风有了新的转变。

　　这些成绩的取得，是省委、省政府亲切关怀、大力支持的结果，是市委总揽全局、坚强领导的结果，是市人大、市政协认真履职、有效监督的结果，也凝聚着全市人民的智慧和汗水。我代表市政府，向全市各族人民，向人大代表、政协委员，向各民主党派、工商联、无党派人士和各人民团体，向驻朝中省直单位、解放军指战员和武警官兵，表示衷心感谢并致以崇高敬意！

　　各位代表，在总结成绩的同时，我们也清醒地看到面临的严峻形势和存在的突出问题。当前，全球经济的复杂性和不确定性依然突出，经济下行压力还在延续，政治生态遭到严重破坏的负面影响尚未完全消除，困难压力前所未有。一些新老问题相互交织，不容忽视。一是经济发展动能不足，导致增长乏力。产业基础薄弱，总量不大、质量不高，改革和创新活力释放还不充分，旧的动能在减弱，新的增长动能尚未形成。二是产业结构单一，典型的“二人转”特征突出，导致经济增长波动大。不仅三次产业结构不合理，产业内部结构也不合理。三是投资营商环境比较优势尚未建立起来，企业、项目生长的环境和土壤不佳。四是政府债务、养老金发放、财政收支等风险不容低估，潜在的社会风险和不稳定因素仍然较多。五是民生保障仍是最大短板，脱贫攻坚任务艰巨，发展中的不平衡、不协调问题仍很突出。六是政府系统党的领导弱化、党的建设缺失、全面从严治党不力问题依然存在，部分政府工作人员精神状态不佳，思想观念落后，不担当、不求实、不勤不廉等问题仍然突出。形成这些问题的原因，有历史的和现实的，也有客观的和主观的，有体制的和具体的，也有思想的和能力方面的，多种因素交织而成。找出问题，目的是为了更深入地分析市情，找到工作短板和努力方向，进而引领新一年工作。

　　2017年工作安排

　　今年是朝阳振兴发展的关键之年，是本届政府的收官之年，也是我们奋力追赶全国经济发展平均水平的冲锋之年。现在，全面建成小康社会进程已进入倒计时，冲刺时间十分宝贵；全面振兴进入滚石上山、爬坡过坎的关键时段，任务更加艰巨繁重；随着经济数据的全面做实，新一年政府的状态、成绩备受关注。我们必须牢固树立危机意识、责任意识和进取意识，以昂扬斗志和实干精神，奋力做好今年工作。

　　朝阳的发展从来都是在越过困境、应对挑战中实现的。只要坚定信心，就没有过不去的坎。我们要看到，中央7号文件、国务院62号文件出台了一系列含金量高、操作性强的重大政策举措，为朝阳振兴发展提供了难得的历史机遇。我们要看到，随着改革开放的不断深化，特别是京沈高铁即将通车，朝阳的固有优势和改革开放红利正在释放，区位条件和发展环境正在发生突变，后发优势日益显著。我们要看到，如今的朝阳，已形成了科学清晰的发展思路，确立了“五个一工程”建设的发展重心，迈开了经济转型升级的步子，一批新的经济增长点正在加快形成。我们要看到，经过政治生态的全面修复，干部的干劲和激情正在提振，干事创业的动能正在集聚。信心就是希望，信心就是勇气，信心就是力量。只要我们坚定信心，苦干实干，再大的困难都阻挡不了朝阳前进的脚步，动摇不了朝阳人民奋勇直前、追逐梦想的决心！

　　做好今年政府工作，要以党的十八大和十八届三中、四中、五中、六中全会精神为指引，深入贯彻落实省十二次党代会和市十次党代会精神，适应把握引领经济发展新常态，持之以恒落实五大发展理念和“四个着力”，坚持稳中求进工作总基调，以推进供给侧结构性改革为主线，坚定不移地落实振兴发展、脱贫攻坚、修复政治生态三大任务，努力提升发展质量和效益，发展速度不低于全国全省平均水平，高于辽西北地区平均水平。

　　主要预期目标是：地区生产总值增长6.5%，力争7%以上；公共财政预算收入增长5%，力争6%以上；固定资产投资增长12%，力争16%；社会消费品零售总额增长8%，力争9%；城镇登记失业率控制在4.5%以内；城乡居民人均可支配收入分别增长7%和8%。

　　确定这样的预期目标，就是要力求在实事求是的基础上，树立更高的标准，自我加压，铆足干劲，一步一个脚印地去苦干实干。

　　一、着力增强发展动力

　　动力是朝阳发展的根基，需要更多更好的项目持续支撑，需要深化改革不断催生，需要创新创业的新动能转换植入，通过多管齐下，不断打造新的发展引擎。

　　一是把上项目作为经济工作的核心和重中之重。项目是解决朝阳发展的根本，唯此为大、唯此为先、唯此为重。目前项目建设的问题还很多，突出表现在：储备不足，可落地的少，支撑不了今年固定资产投资任务所需的增量；准备开复工的项目尚不托底，要素保障还未解决到位；一些干部做实项目的意识差，习惯于“笼而统之”，在数字之间“游走”；有的地区和部门危机感、压力感不强，抓项目的狠劲、韧劲和实劲不足。我们要对准这些病灶，创新推进机制和考核办法，尽快取得突破。

　　1.持续抓好项目年。坚持市县四大班子一齐抓，一二三产业一齐抓，国资民资外资一齐抓，增量和存量一齐抓，特别是大中小项目一齐抓，既盯住大项目，也不放过能带动就业、增加税源的中小项目，使项目建设既有“巨轮引航”，也有“千帆竞发”。更多地包装、策划和储备项目，更实地推动项目尽快签约落地、开工建设、投产达效。重点做实“两个库”：一个是“年度开复工项目库”。对年内准备开复工的229个项目，特别是155个亿元项目实行分类入库，逐一梳理审批、融资、拆动迁等要素保障问题，建立实名制台帐，明确办理责任和时限，利用开春前三个月集中搞好要素保障，并形成长效管理机制。开复工项目库要实行动态管理，不断补充新项目。另一个是“年度招商项目储备库”。进一步更新招商理念，设立招商产业图谱，建立招商项目、推进项目、落地项目“三库一体”储备方式。精心谋划包装，千方百计招商，最大限度寻求项目资源。同时建立项目评估考核体系，项目落地后及时转入“年度开复工项目库”，弥补当年项目投资不足。

　　做实“两个库”的前提是实事求是，把任务指标分解到县（市）区和园景区、分解到部门、分解到人头，层层实行项目实名制，严格进行调度督查和通报问责，决不能“玩数字”、弄虚作假。

　　2.提升“五个一工程”。“五个一工程”既是引领，又是载体，更是抓项目搞服务的实体。要巩固良好的发展态势，把各园景区建设成引领性更强、体制更新、吸引力更大、政策更好的龙头工程和项目载体，在谋划发展方向、园区规划、破解融资、基础设施建设、管理体制、软环境建设、服务水平以及政策比较优势和项目集聚等方面，争取新的更大的突破。提升“五个一工程”必须解放思想，破除旧观念，善于创新，敢于创造，把一切资源和力量向各园景区聚焦集聚。

　　3.盘活存量企业资源。抓存量也是扩大投资。尽管我市存量企业发展遇到了困难，但仍是经济的现实支撑。要逐户排查存量企业减产停产原因，分类施策，加强服务，围绕生产经营困难企业抓解困帮扶，围绕本地产品上下游配套抓对接帮扶，围绕矿业整顿后的47户铁选企业抓精准帮扶，帮助企业复产、达产、扩产。继续抓好盘活存量土地、厂房工作。实施民营工业企业“大做强”“小升规”“个转企”三项工程。规模以上工业增加值增长7%，力争8%。

　　4.扩大对外开放。主动融入京津冀协同发展战略，积极开展产业集群招商和以商招商，同时抓好以周边国家、地区和欧美为重点的国外招商，引进内资和外资均增长10%。积极面向“一带一路”沿线国家以及非洲开展国际产能合作，加大出口企业支持力度，培育壮大出口基地，外贸出口在全省所占份额不低于1.1%。

　　5.主动争取重大政策。抢抓中央7号文件、国务院62号文件等政策机遇，在争取产业衰退地区转型发展试点、老工业基地转型升级示范区、通用航空产业综合示范区，以及争创国家级高新技术产业开发区上力求突破。

　　此外，还要未雨绸缪，高度重视高铁开通带来的机遇和挑战，用更长远、更开放的眼光，提前抓好“高铁时代”谋划和准备工作，掌握发展主动权。

　　二是深化改革激发内生动力。朝阳的困难和问题，深层次都源于体制机制落后，积弊较深，归根结底要靠深化改革来破解，向改革要动力、要资源、要突破。

　　1.深化“放管服”改革。进一步简政放权，市县同步、全域推进全国行政审批制度改革试点，推动行政审批和市场监管工作高效运行。深化商事制度改革，全面推行“五证合一、一照一码”“先照后证”和个体工商户“两证整合”，放宽市场准入。以信用监管为核心，推进“双随机一公开”监管改革全覆盖，全面实行涉企信息统一归集公示，建立失信联合惩戒机制，打造“诚信朝阳”。抓好重点园区企业投资项目承诺制试点工作，并探索“零审批”园区试点，推进园区封闭运行。

　　2.深化国资国企改革和事业单位分类改革。坚持一盘棋思想，加快并优化水务、城发、文投集团和资本运营公司运行和发展，推进具备条件板块组建新的国有企业集团，力求在统筹盘活全域优质资产资源上取得新的更大进展。坚决打好国企提质增效攻坚战，选择1—2家国有企业开展混合所有制改革试点。全面解决“三供一业”分离移交和厂办大集体遗留问题。加快推进生产经营类事业单位转企改制。实施事业单位公车改革。

　　3.深化农村改革。扩大农业共营制改革试点覆盖面，选择1—2个县整体推进。全面开展农村土地确权颁证，推动土地承包经营权有序流转。启动农村综合产权交易市场建设。继续深化供销社综合改革，完成国有林场改革。

　　三是激发创新创业活力。朝阳发展新动力归根结底要靠创新驱动，我们必须摆脱旧的观念束缚和路径依赖，让创新创业的种子在朝阳大地生根发芽、开花结果。

　　1.支持科技创新。加强与高校和科研院所的深度合作，组建产业技术创新联盟，重点扶持1—2个创新型园区和一批创新型企业，引领和促进科技成果引进、消化、吸收和再创新，实施科技成果转化项目50个以上，高新技术企业数量、质量实现“双提升”。

　　2.推动大众创业、万众创新。巩固完善众创空间、创新工坊、企业孵化器等载体建设，提高孵化基地运营服务质量，建设一批省市级双创示范基地。落实创业担保贷款、税费减免、创业培训补贴等政策，让有梦想、有意愿、有能力的市场创业主体“如鱼得水”，施展才华。

　　3.抓紧解决人才短板。围绕“五个一工程”建设、国有企业集团组建，积极引进高端人才和团队。探索整合优质职教资源，加快推进朝阳师专向高职学院转型，建立适应本地发展需求的现代职业教育体系。命名表彰一批市级优秀企业家、杰出工程师、朝阳技师和乡土能工巧匠。

　　二、着力调整产业结构

　　加快产业结构调整、实现经济转型，是摆在朝阳发展面前最大的难题。这不是短期能实现的，我们必须保持定力，卧薪尝胆，一步一个脚印地补好这堂课。

　　一是落实好“三去一降一补”任务。朝阳经济循环不畅，很重要的表现是实体经济供需失衡。必须坚持从供给侧上想办法，把握好“进”与“退”的关系，继续化解钢铁、煤炭过剩产能，抓好矿山矿业整治，加快矿产资源开发向大企业集中、向精深加工转型。继续消化房地产库存，实施棚改1.29万户，商品房销售面积增长10%。加强和改进金融服务，积极争取国家专项建设基金，设立产业投资引导基金，推进债转股，用好多样化的金融工具推动产融合作，降低企业杠杆率。继续在降低企业税费负担、要素和交易成本上打好“组合拳”。抓住关键环节，聚焦补齐各种发展短板。

　　二是加快推进工业转型升级。工业以矿为主、“一铁独大”的畸形结构，是朝阳发展的突出症结。受市场变化影响，工业实体经济萎缩问题也越发凸显。要抓住工业结构调整不撒手，全面落实中国制造2025朝阳行动纲要，用新技术新业态改造提升冶金、汽车及零部件、建材等传统产业。支持凌钢搞好优特钢研发生产，在抓好主业的同时大力发展非钢产业；支持一汽凌源汽车生产、飞马智能化技改、浪马轮胎技改等一批重点项目。加快培育战略性新兴产业，实施制造业智能升级三年行动计划，支持新能源汽车、新能源及高端装备、新材料、节能环保、电子信息等产业发展，点燃未来工业新的希望之光。

　　三是加快搞好农业供给侧结构调整。农业是朝阳的最大优势。但目前“原”字号痕迹重，农产品加工和品牌建设相对滞后，市场和流通体系建设还不够完善。我们必须下力量调好调顺调优农业结构，重点抓好农产品加工园区基础设施和重点项目建设。深入实施设施农业扩量提质增效工程，加快调减玉米种植面积，大力发展杂粮、蔬菜、花卉、食用菌、中药材等高效作物，优化粮经比例。充分利用国家实施北方农牧交错带农业结构调整政策，大力发展畜牧业，抓好国家生猪、奶牛种养结合整县推进试点。注重农产品质量安全，推进“朝阳小米”“朝阳蔬菜”区域公用品牌创建，抓好高效节水灌溉工程，提高人工增雨能力，加快农产品现代流通体系和区域集散中心建设，促进农业向提质增效、可持续发展转变。

　　四是加快发展现代服务业。近年来我市服务业总体发展比较快，但现代服务业基础差、总量小，特别是丰富的文化旅游资源尚未得到充分开发利用。这既是劣势，也是方向和潜力。要加快推进服务业集聚区建设，抓好高铁商务区、万达商圈、北票农产品物流园等重点项目。加快传统业态转型升级，大力发展电子商务、信息、金融、会展、现代物流、养老养生、体育健身等新兴产业，打造新的消费热点。积极引进和新建金融机构，引导企业到多层次资本市场融资，完成融资140亿元，新增金融机构3个、各类股权交易市场挂牌企业6户。推进朝阳银行经营转型与创新，巩固拓展沈阳、大连等外埠市场。修编朝阳旅游发展规划，探索旅游文化体育等相关产业多规整合办法。组建文旅集团，建立旅游文化产业基金。启动“三燕文化街区”等重点项目建设，推动牛河梁、热水汤等七大景区对外合资合作，打造旅游品牌。

　　五是加快实现新型城镇化。同步抓好老城区改造提升、新城区设施配套和产城融合发展，形成以中心城市为核心、以县城为基础、以新市镇和特色镇为支点的城镇建设新格局。每个县（市）区在抓好新市镇试点的基础上，再抓好一批重点镇和特色镇建设。稳步推进农业转移人口市民化，进一步提高户籍人口城镇化率。

　　三、抓好软硬环境建设

　　环境是经济发展的磁场，只有让软环境更硬、硬环境更优，“软硬兼修”，一个地区发展才会有竞争力。

　　一是全面优化营商环境。下决心下力量营造崇尚企业、崇尚纳税人的社会环境，在体制、理念和抓法上寻求大的突破，打造明显的区域软环境比较优势。大力开展软环境建设年活动，认真落实《辽宁省优化营商环境条例》，探索强化软环境建设领导体制和工作机制，成立市营商环境监督管理局，逐步延伸其监管处置职能。复制推广国家和省前沿性政策、改革模式和探索性经验，分层次谋划制定特色招商政策，营造新常态下的政策优势。坚决取缔没有法定依据的各种审批审查、投资限制、资格认证、中介服务、行政收费。全面推行重点项目首问负责、代理代办服务。抓住突破软环境建设困局的关键点，集中开展专项整治活动，构建“亲”“清”政商关系。

　　二是搞好基础设施建设。做好京沈高铁朝盘线征地拆迁工作，协调推进高铁五座站房和喀赤线、锦承线、叶赤线朝阳段项目建设。加快环城生态绿廊、北部城区集中供热、辽西北供水配套净水厂及配水管网建设，启动高铁北站交通枢纽、文体路等重点工程，全面完成朝马线公铁立交项目。

　　三是打造一流生态宜居环境。坚决贯彻“气十条”“水十条”“土十条”，深入推进青山、碧水、蓝天、沃土工程，实现造林绿化60万亩、水土保持生态治理70万亩；出重拳防治大气污染、治理工业污染，做好城中村和城边村改造、淘汰各类燃煤小锅炉等工作，空气质量继续保持全省前列；全面推行河长制，加强凌河水质保护，实施城乡污水治理提标改造、农药化肥减施、土壤污染防治等工程。推进生态市（县）创建工作，开展农村环境综合整治活动。坚持建管并重，实施城市智慧化管理，切实解决交通拥堵、车辆占道停放、乱搭乱建等人民群众反映强烈的问题，打造优美整洁、管理有序的城市环境。

　　四、抓好各种风险管控

　　新常态伴随着新问题新矛盾。在抓发展的同时，必须增强忧患意识，居安思危，积极应对各种风险考验。

　　一是积极化解政府债务。我市政府债务总额比较大，尽管已置换了当期和逾期债务，但只是缓兵之计。我们必须认真贯彻国务院88号文件和辽委发21号文件精神，增强债务管控意识，建立健全风险应急处置机制，严格控制新增债务，积极处置化解存量债务。既要科学有效管控好债务风险，又要想方设法为地区发展腾挪出充分的融资空间。

　　二是保证养老金及时足额支付。这项工作事关朝阳改革发展稳定大局，事关百姓福祉。要认真落实辽委发19号文件精神，按照精算平衡原则，严格加强养老金收支管理，确保按时足额发放。

　　三是强化财政收支管理。继续推进财税体制改革，加强预算管理，强化收入征管，优化支出结构，压缩一般性支出，保障民生等重点支出。

　　四是全力维护社会稳定。深入做好多元化矛盾纠纷排查化解，实现阳光信访、责任信访、法治信访。健全立体化、信息化社会治安防控体系，深化平安创建活动，争创全国综治优秀市。依托“互联网+公安”综合信息服务平台，提高服务发展新质态。抓好安全生产责任制落实和重点领域常态化隐患排查治理，坚决杜绝重特大事故发生。强化交通、消防基础设施建设，提高防灾救灾能力。严格落实食品药品安全制度。扎实做好政务公开和市民诉求办理工作。

　　五、着力保障和改善民生

　　越是在困难情况下，越要重视民生。我们要继续收紧开支，把有限的钱花在民生工程上，用在解民忧、惠民生、暖民心上，牢牢守住民生底线。

　　一是坚决打赢脱贫攻坚战。精准脱贫是一项重大的政治任务，我们立了军令状、做了承诺，就必须紧盯不放，一抓到底。开拓思路，创新方法，建立更科学、更符合实际的贫困退出标准和动态考评机制。充分利用“扶贫贷”撬动银行贷款和社会资本。进一步扩大产业扶贫覆盖面，把更多的贫困人口纳入产业扶贫链条。强化医疗扶贫、教育扶贫、低保兜底作用，积极探索生态扶贫等新模式，全面完成5.27万贫困人口脱贫、115个省级贫困村和71个市级贫困村销号任务。我们坚决不搞层层加码，不搞提前完成任务，集中心思、脚踏实地地巩固、扩大、做实、做稳扶贫脱贫工作。

　　二是抓好就业和民生保障。大力开展订单式技能培训，启动市级公共实训基地建设，打造国家级标准公共就业服务平台，城镇新增就业1.6万人。夯实“五险合一”，推进省内异地就医即时结算，做好退休人员基本养老金、城乡低保、城镇居民医疗保险和新农合补助标准提标工作。建立让市民参与的供暖质量评价机制和失信供暖企业退出机制。推进不动产统一登记工作。加强大病救助和困难家庭临时救助，发展慈善和残疾人事业。

　　三是协调发展社会事业。新建改扩建标准化幼儿园10所，加快实施百所义务教育学校“全面改薄”，继续做好义务教育发展基本均衡县（区）国家和省级验收工作。推进城市公立医院改革，加快分级诊疗和家庭医生签约服务制度建设，实现新农合省内异地就医即时结算，推进贫困村卫生室全覆盖。创新公共文化服务体系建设模式，在贫困村率先推广“综合文化服务中心”建设新标准，并实现全覆盖。加大牛河梁红山文化遗址“申遗”工作力度，积极争创历史文化名城。开展“十大品牌”群众体育活动，提升竞技体育综合实力。全面落实民族宗教政策，加快民族地区社会事业发展。大力开展双拥共建活动，抓好国防动员、国防教育、人民防空工作，支持工会、共青团、妇联等人民团体发挥作用，做好外事侨务、档案等工作。

　　六、加强政府自身建设

　　一是加强政府系统党的建设，把从严治党要求内化为推动工作的强大动力。坚决整治党的领导弱化、党的建设缺失、从严治党不力问题，认真履行“一岗双责”，抓实党风廉政建设和反腐败工作。把党建成果尽快转化为推动工作的动力，向政府系统的思想建设、作风建设、业务建设全面延伸拓展。

　　二是坚持依法行政，把政府工作全面纳入法治轨道。主动接受人大及其常委会的法律监督、工作监督和政协的民主监督，认真执行人大及其常委会决议决定，积极办理人大代表建议和政协提案，做到件件有着落、事事有回音。做好矿山植被恢复和水资源管理立法相关工作。

　　三是解放思想，敢于担当，提高执行力。对标先进地区，解放思想，大胆创新，积极进取，干事创业。健全容错纠错机制，旗帜鲜明地为敢于担当的干部担当，为敢于负责的干部负责。

　　四是发扬“钉钉子”精神，狠抓落实。坚持查问题、找原因、明举措、抓落实，从严整治工作忽悠、弄虚作假、作风漂浮等问题，做实责任主体、落实工作措施，加大督查问责和考核奖惩力度，形成抓铁有痕、踏石有印的工作氛围，凝聚振兴发展正能量。

　　各位代表，行动是最好的语言。新的一年，我们将在市委的坚强领导下，凝聚全市各族人民的智慧和力量，以更高的标准、更强的举措、更实的作风，攻坚克难，苦干实干，向着全面建成小康社会和实现朝阳振兴发展的宏伟目标奋勇前进，以优异成绩迎接党的十九大胜利召开！
